# Health condition, income loss, food insecurity and other social inequities among migrants and refugees during the COVID-19 pandemic in Brazil

**DOI:** 10.1186/s12889-023-16620-9

**Published:** 2023-09-05

**Authors:** Heriederson Sávio Dias Moura, Thaís Zamboni Berra, Rander Junior Rosa, Ruan Víctor dos Santos Silva, Débora de Almeida Soares, Juliana Soares Tenório de Araújo, Fernanda Bruzadelli Paulino Costa, Rosa Maria Pinheiro de Souza, Murilo César do Nascimento, Titilade Kehinde Ayandeyi Teibo, Maria Del Pilar Serrano-Gallardo, Ricardo Alexandre Arcêncio

**Affiliations:** 1 Avenida dos Bandeirantes, University of São Paulo at Ribeirão Preto School of Nursing, São Paulo, Brazil 3900, Monte Alegre, Ribeirão Preto, 14040-902; 2 Institute of Hygiene and Tropical Medicine, New University of Lisbon, Lisbon, Portugal Rua da Junqueira, 100, 1349-008; 3 National School of Public Health Sergio Arouca, Oswaldo Cruz Foundation, Rio de Janeiro, Brazil; 4 Federal University of Alfenas, Minas Gerais, Brazil; 5 Autonomous University of Madrid, Madrid, Spain

**Keywords:** COVID-19, Migrant population, Pandemic, Transients and Migrants

## Abstract

**Background:**

Brazil is the destination of many international migrants and refugees and, given the circumstances of their entry into the country, many face difficulties due to the absence of targeted policies. Thus, the objective of this study was to survey the social impact of COVID-19 on international migrants and refugees regarding income loss, food insecurity and other social inequities, and to identify explanatory factors on these aspects.

**Methods:**

This is a cross-sectional, analytical study. We used a validated instrument applied by trained interviewers. Descriptive analysis and binary logistic regression were performed to identify factors associated with income loss and food insecurity.

**Results:**

A total of 360 individuals from sub-Saharan African and South American countries participated in the study. Individuals who were white, black/brown, yellow, had an occupation/employment, and earned less than one minimum wage were more likely to lose income. Those who reported no income, received less than one minimum wage, and were diagnosed with COVID-19 were more likely to be food insecure.

**Conclusions:**

The study advances knowledge by identifying factors associated with income loss, food insecurity, and individuals' difficulty in accessing health services and social support measures in Brazil.

## Background

The health crisis caused by the COVID-19 pandemic provoked unprecedented impacts on society, highlighting numerous policy weaknesses in various countries in terms of social, economic, health, and other sectors. In this context, the social protection systems of some Latin American countries were the most affected, as they were overwhelmed by the number of displaced people, which grew by 400% in the last 10 years within this territory, due to international migratory movements motivated by unemployment, poverty, hunger, and other situations of social vulnerability [1].

The term International Migration is defined as “*Movements of people who leave their countries of origin or habitual residence to settle, permanently or temporarily, in another country. Consequently, it implies the crossing of international borders*” [2]. In 1951, the United Nations Convention defined a refugee as “*any person who, owing to a well-founded fear of persecution on account of their race, religion, nationality, membership of a particular social group, or political opinion, remains outside their country of origin, and because of these fears, they cannot or will not return to their country*” [3]. The migratory phenomenon is in an active process of evolution and this becomes a permanent challenge for the implementation of global actions aimed at protecting international migrants and refugees, who are in a constant situation of risk and vulnerability [4].

Previous studies point out that, despite Brazil's pioneering role in the formulation of laws and protective social measures aimed at ensuring equal rights for international migrants and refugees in its territory, the implementation of these actions have not kept pace with the dynamics of migration and the consequent worsening of social vulnerabilities that this phenomenon causes, when it occurs in a disorderly manner [1].

During the displacement process, it is common for international migrants and refugees to be subjected to stressful and even traumatic situations, since the difficulties begin when they leave their countries of origin, often leaving behind family members and loved ones, migrating under unhealthy conditions of transportation, hunger, and conditioned to pay high fees for displacement. Such situations become even more accentuated when they arrive in the countries to which they are moving, as they face difficulty in accessing basic goods and services such as food, housing, and health services [5, 6].

It is also noted that the migration processes during the pandemic brought increasing concerns regarding working conditions, as the work activities of international migrants and refugees have a high turnover and low wages [7]. In these conditions, there are difficulties in relation to income and basic human needs, such as food, since international migrants and refugees find themselves without the social protection and labor rights that come from formal employment [8].

Under these conditions, food insecurity becomes acute and affects natives as well as international migrants and refugees. By the end of 2020, about 19.1 million Brazilians were living with hunger, and by 2022, an estimated 33.1 million people did not have enough to eat or have enough food [9].

Several studies have addressed the social impact brought and exacerbated by the COVID-19 pandemic, especially on international migrant and refugee populations around the world [10–12], however, studies that have focused on this theme in the Brazilian context are still incipient.

Although the country has stood out in the discussion on inclusive policies that can mitigate the social effects of the pandemic, especially among international migrants and refugees, in practice it is not known how much this population was affected, which lacks an investigative approach with this focus.

We emphasize that in the present study, we considered food insecurity the financial difficulty that this population had to buy food during the pandemic, so that measurement scales were not used. Thus, the objective of this study was to assess the social impact of COVID-19 on international migrants and refugees in terms of income loss, food insecurity and other social inequities, and to identify explanatory factors on these aspects, providing a scope of evidence for affirmative actions, health policies and social protection.

## Methods

### Study design

This is a cross-sectional [13, 14], analytical study, carried out by means of field interviews conducted from May 2022 to March 2023. This study stems from a matrix survey entitled “Social Thermometer—COVID-19 in Brazil”, which has as its unit of analysis the capital cities of Brazil.

Regarding its location, Brazil is located in the American Continent, specifically in South America, and is considered the fifth largest country in the world in land area, with a land area of 8,515,767 km^2^ [15], the third largest country in the American Continent and the largest country in the South American territory, occupying almost 50% of its total area. Brazil shares borders with 10 of the 12 South American countries: Argentina, Bolivia, Colombia, French Guiana, Guyana, Paraguay, Peru, Suriname, Uruguay and Venezuela; only Chile and Ecuador do not have borders [16].

### Population and samples

The matrix project had, as one of its target populations, people in situations of social vulnerability, namely: individuals living in slums, camps for internally displaced people, homeless individuals, and international migrants/refugees. At least one of these groups was recruited in each capital of the 26 federative units and the Brazilian Federal District. For the present study, we are considering only international migrants and/or refugees.

We included those who understood the language spoken in Brazil (Brazilian Portuguese), had been residing for at least six months in the country and were 18 years of age or older. We excluded those who did not fully answer the research questionnaire.

Due to the characteristics of the studied population, not being included in official records in Brazil, especially regarding those in irregular situations and also due to confidentiality concerns, participant recruitment was carried out using a non-probabilistic technique of sequential sampling, where participants were included as they were located and agreed to participate in the study [17]. Even so, the calculation for finite populations was used, according to the following parameters: confidence level 95%, random sampling error 5%, power of the test 80%, and variance 50%, since we do not know the event, with an additional 10% referring to possible losses, conforming a minimum sample of 360 people.

### Instruments and data collection

The instrument used in the study, entitled “COVID-19 Social Thermometer: Social Opinion”, was adapted and validated for Brazil through the Delphi technique [18], from an instrument developed and validated by researchers at the National School of Public Health at the New University of Lisbon (ENSP/UNL) in Portugal, and published in studies to assess risk perception [19], behavioral patterns [20] and adherence to protective health measures [21].

The instrument was developed on the REDCap platform [22, 23] at the University of São Paulo (USP) Ribeirão Preto campus. REDCap is a browser-based, metadata-driven electronic data capture (EDC) software with a workflow methodology for designing clinical and observational research databases. The instrument consists of 141 fields, containing questions in checklist format, multiple choice and Likert-type scale.

For the application of the instrument, a network of contacts was created for recruitment of participants through professionals linked to research institutions, universities and civil society actors for application of the instrument. We also adopted the strategy of mobilizing participants through Social Movements (SM), due to the fact that they are exponents for the link to the territories and to the populations in situations of social vulnerability due to the interlocution created through the various key strategies developed in these locations and to these populations.

The instrument was applied by field interviewers enrolled in the survey, using cell phones and/or tablets, and the average application time was 20 to 30 min. The interviewers were also trained to apply the instrument in order to avoid measurement bias. The interviewers were instructed to consider the pandemic period as of March 11, 2020, when the World Health Organization (WHO) issued an alert for a pandemic situation caused by COVID-19 [24, 25], until the time of the interview. To start the questionnaire application, the Informed Consent Form (ICF) was read to the study participants and, only after agreement and signature, was the interview started. The ICF was structured in two copies, which were read in their entirety, by the participant and the responsible interviewer, and the application of the instrument followed only once for each person. In the cases of illiterates, fingerprints were collected, each party having one copy, and the interviewer informed the participant of the importance of keeping the document. It is worth mentioning that participation in the research was voluntary.

### Statistical analysis

After consistency analysis and database standardization, initially, aiming to characterize the study participants, descriptive analyses were performed with calculation of absolute frequency (n) and relative frequency (%) measures, according to sociodemographic, clinical, and pandemic-related variables of COVID-19. Data were tabulated in Microsoft Office Excel 2010 spreadsheets, imported and analyzed using R version 4.1.1 software.

Then, in order to know the origin of the study participants, a flow map was built from the geographic coordinates (latitude and longitude) of the centroid of the country of origin and the geographic coordinates of the centroid of the destination country (Brazil), using the ArcGis 10.5 software.

Subsequently, six variables of interest were selected, namely: has occupation/employment (yes or no), has housing (yes or no), receives some type of government assistance (yes or no), uses the Unified Health System (SUS – *Sistema Único de Saúde*) (yes or no), lost income during the COVID-19 pandemic (yes or no), had or has financial difficulty in acquiring food during the COVID-19 pandemic (yes or no). The answers to these variables were dichotomized and grouped according to the Federative Units (FU) of residence of the research participant, in order to visualize in which Brazilian regions the international migrants and/or refugees presented greater situations of social vulnerability. The variables were organized in Microsoft Office Excel 2010 spreadsheets and then maps were built with the information through ArcGis 10.5 software, using the shapefile provided by Brazilian Institute of Geography and Statistics (IBGE – *Instituto Brasileiro de Geografia e Estatística.*) [26].

Finally, to identify the factors associated with the outcomes of interest, binary logistic regression was used based on the variables present in the “COVID-19 Social Thermometer: Social Opinion” instrument. For better understanding, the variables under study in this analysis were grouped into:

- Independent variables

As independent variables for both outcomes of interest we considered: Gender (Male, Female, Transgender person); Race/Color (Black/Brown, White, Yellow, Indigenous, No declaration); Age (From 18 to 29 years old, 30 to 59 years old, 60 years or older); Marital Status (Single, Married or in a stable union, Divorced or legally separated, Widowed); Occupation/employment (Unemployed, Informal/secret, Self-employed, Private employee, Micro Entrepreneur, Public employee, Student, Domestic employee, Entrepreneur, Farmer Family, Others); Resides in a territory of social vulnerability (Yes, No); Housing (Shelter, Rended, Owned, Sold, Street, Other); Schooling (Secondary incomplete or complete, Elementary School incomplete or complete, College incomplete or more, No schooling); Monthly family income (No income, Less than 1 minimum wage, From 1 to 2 minimum wages, I prefer not to inform, Above 3 minimum wages, I do not know, From 2 to 3 minimum wages, No Answer); Receives some government assistance (Yes, No); Uses SUS (Yes, No); Has a Chronic illness (Yes, No, No Answer); Compared to before COVID-19, how have you been feeling most of the time? (I am more agitated, anxious or tense (Yes, No); I am more irritable (Yes, No); I am more sad, more easily discouraged or cry (Yes, No); I am more lonely (Yes, No)); and Have you had a confirmed diagnosis of COVID-19 before or after the vaccine (Yes, No).

- Dependent variables

Two binary logistic regressions were performed, so that the first dependent variable considered: international migrants and/or refugees who lost income during the COVID-19 pandemic, which was dichotomized into 0 (did not lose or had no income) and 1 (lost partially or totally lost income). For the second dependent variable, we considered: migrants and/or international refugees who have or had financial difficulties in acquiring food during the COVID-19 pandemic, which was also dichotomized into 0 (had financial difficulties or never had financial difficulties in acquiring food) and 1 (did not have financial difficulties and now has or always had financial difficulties to purchase food).

Initially, an exploratory analysis was conducted to verify the presence of multicollinearity between the independent variables that were tested using the Variance Inflation Factor (VIF), and those with values greater than 10 [27] were not included in the complete model.

Modeling was carried out using the Backward method, which starts with a complete model (with all variables, with the exception of those with VIF > 10) and then removes the variables one by one and checks the behavior of the model. The best model considered was the one with the lowest value of the Akaike Information Criterion (AIC) [28]. For the final model, the Odds Ratio (OR) were calculated for the statistically significant variables (*p* value < 0.05) with their respective 95% Confidence Intervals (95%CI). It is noteworthy that all categories of independent variables were dichotomized (0 and 1) to be inserted in the logistic regression model, so that all categories were tested and compared with the others, without the need to choose a reference category or perform OR adjustment.

After choosing the final model based on the lowest AIC value, the Hosmer–Lemeshow, likelihood ratio, CoxSnell, Nagelkerke and McFadden tests were performed to validate the model. Furthermore, the predictive capacity and accuracy of the models were verified based on the area under the Receiver Operating Characteristic (ROC) curve with their respective 95% CI values [29]. The analyses regarding logistic regression and validation tests were performed using RStudio software, version 4.1.1.

## Results

A total of 360 individuals participated in the study in Brazil, including international migrants and/or refugees. It was observed that two participants were from Sergipe, 13 from Pará, 100 from Roraima, 28 from Rio Grande do Sul, three from Mato Grosso, one from Santa Catarina, 57 from Goiás, two from Paraíba, one from Amapá, 12 from Alagoas, 31 from Amazonas, two Rio Grande do Norte, 11 from Rondônia, 14 from Acre, 29 from Maranhão, 21 from São Paulo, and five from Piauí. Most of whom were male (*n* = 207; 57.5%), of black or brown race/color (*n* = 205; 56.9%), aged between 30 and 59 years (*n* = 248; 68.9%), single (*n* = 195; 54.2%), unemployed (*n* = 174; 48.3%), residing in a territory of social vulnerability (*n* = 338; 93.9%). The main housing was shelters (*n* = 160; 44.4%). About education, most had incomplete or complete secondary school (*n* = 173; 48.1%), no income (*n* = 150; 41.7%) or income less than 1 minimum wage (*n* = 113; 31.4%), did not receive government assistance (*n* = 304; 84.4%), used SUS (*n* = 333; 92.5%) and had a chronic disease (*n* = 314; 87.2%) (Table 1).

**Table 1:** Sociodemographic and clinical characteristics, loss of income, and financial difficulty in acquiring food during the COVID-19 pandemic in Brazil, 2022–2023 (*n* = 360)

Variables	n	%
**Gender**		
Male	207	57.5
Female	151	41.9
Transgender person	2	0.6
**Race/Color**		
Black/Brown	205	56.9
White	52	14.4
Yellow	51	14.2
Indigenous	42	11.7
No declaration	10	2.8
**Age**		
From 18 to 29 years old	104	28.9
30 to 59 years old	248	68.9
60 years or older	8	2.2
**Marital Status**		
Single	195	54.2
Married or in a stable union	147	40.8
Divorced or legally separated	13	3.6
Widowed	5	1.4
**Occupation/Employment**		
Unemployed	174	48.3
Informal/secret	57	15.8
Self-employed	42	11.7
Private employee	37	10.3
Micro Entrepreneur	20	5.6
Public employee	7	1.9
Student	7	1.9
Domestic employee	2	0.6
Entrepreneur	1	0.3
Farmer Family	1	0.3
Others	12	3.3
**Resides in a territory of social vulnerability**		
Yes	338	93.9
No	22	6.1
**Housing**		
Shelter	160	44.4
Rented	154	42.8
Owned	23	6.4
Sold	15	4.2
Street	7	1.9
Other	1	0.3
**Schooling**		
Secondary incomplete or complete	173	48.1
Elementary School incomplete or complete	92	25.6
College incomplete or more	80	22.2
No schooling	15	4.2
**Monthly family income**		
No income	150	41.7
Less than 1 minimum wage	113	31.4
From 1 to 2 minimum wages	51	14.2
I prefer not to inform	17	4.7
Above 3 minimum wages	12	3.3
I do not know	9	2.5
From 2 to 3 minimum wages	7	1.9
NA^a^	1	0.3
**Receives some government assistance**		
Yes	56	15.6
No	304	84.4
**Uses SUS**		
Yes	333	92.5
No	26	7.2
NA^a^	1	0,3
**Has a chronic illness**		
Yes	314	87,2
No	45	12.5
NA^a^	1	0.3
**Lost income during the COVID-19 pandemic**		
Had no income	102	28.3
Partially lost	91	25.3
I totally lost	91	25.3
Did not lose	67	18.6
I prefer not to answer	8	2.2
NA^a^	1	0.3
**Did you have or do you have financial difficulty to buy food during the pandemic of COVID-19?**		
I have always had financial difficulty	159	44.2
I had no financial difficulty and now I have	77	21.4
I never had financial difficulties	58	16.1
I had financial difficulty and no longer have any	43	11.9
I prefer not to answer	23	6.4
**Compared to the period before COVID-19, how have you felt most of the time?**		
**I am more agitated, anxious or tense**		
No	283	78.6
Yes	77	21.4
**I am more irritated**		
No	310	86.1
Yes	50	13.9
**I am sadder, more discouraged, or cry more easily**		
No	296	82.2
Yes	64	17.8
**I am lonelier**		
No	310	86.1
Yes	50	13.9
**Had a confirmed diagnosis of COVID-19 before or after the vaccination**		
No	281	78.0
Yes	79	22.0

^a^*NA* No Answer

Most participants reported having no income when the COVID-19 pandemic started (*n* = 102; 28.3%) and that they had always had financial difficulties to buy food (*n* = 159; 44.2%), considering self-report about the period before and during the pandemic. They reported that compared to the period before COVID-19, they did not feel more agitated, anxious, or tense (*n* = 283; 78.6%), nor more irritable (*n* = 310; 86.1%), nor more sad, discouraged, or crying more easily (*n* = 296; 82.2%), or more lonely (*n* = 310; 86.1%). Most reported no positive COVID-19 diagnosis before or after taking the vaccine (*n* = 280; 78.0%) (Table 1).

**Fig. 1:**
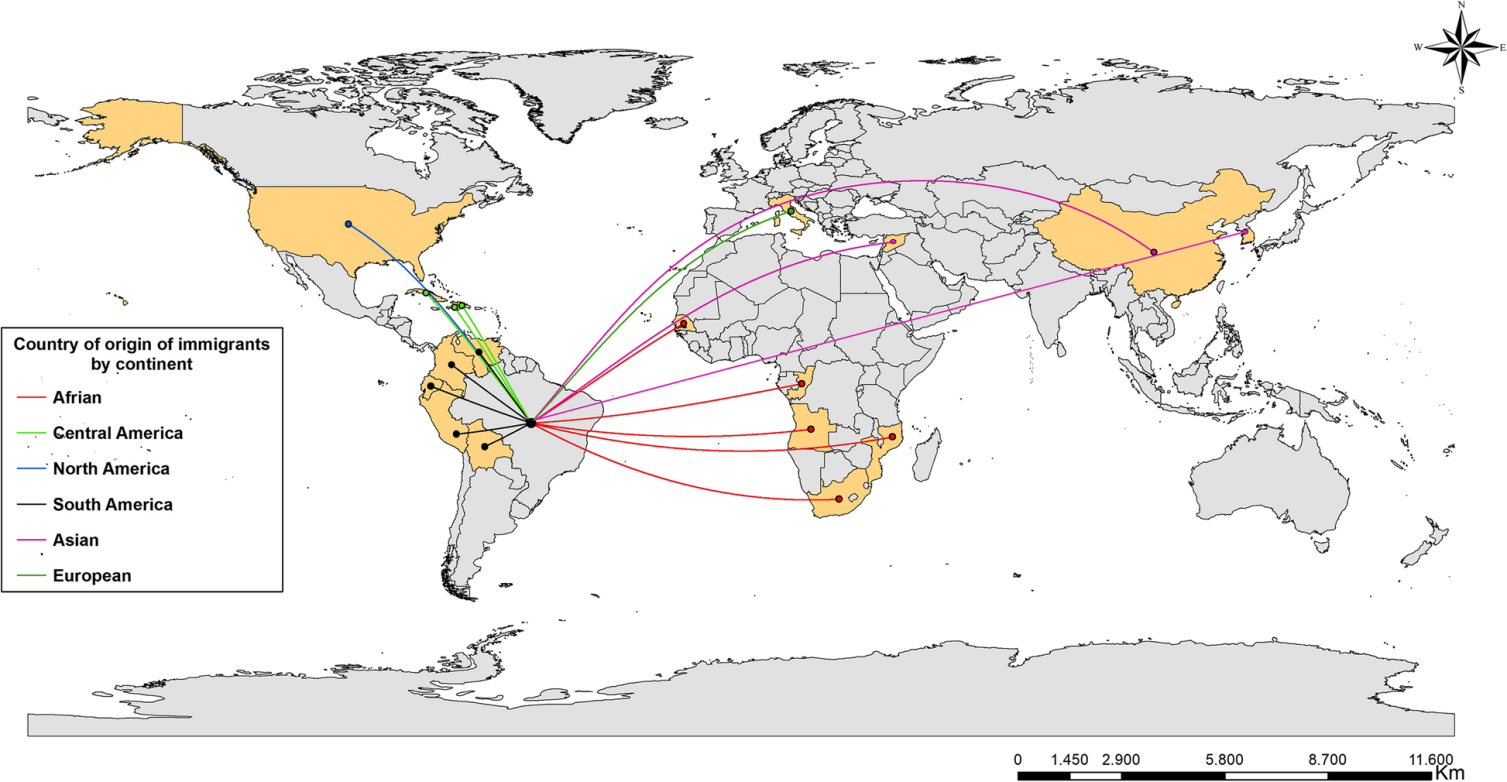
Flow chart of the movement of international migrants and/or refugees to Brazil, considering the reported nationality, Brazil (2022–2023)

The flow chart shows the countries of origin of the international migrants and/or refugees before they moved to Brazil (Fig. 1). It was found that most individuals had sub-Saharan African and South American countries of origin.

Among the populations in situations of social vulnerability interviewed in Brazil in this study, in Bahia, Ceará, Distrito Federal, Espírito Santo, Minas Gerais, Paraná, Pernambuco, Rio de Janeiro, Rio Grande do Sul and Tocantins, there was no record of participation of international migrants and/or refugees.

**Fig. 2:**
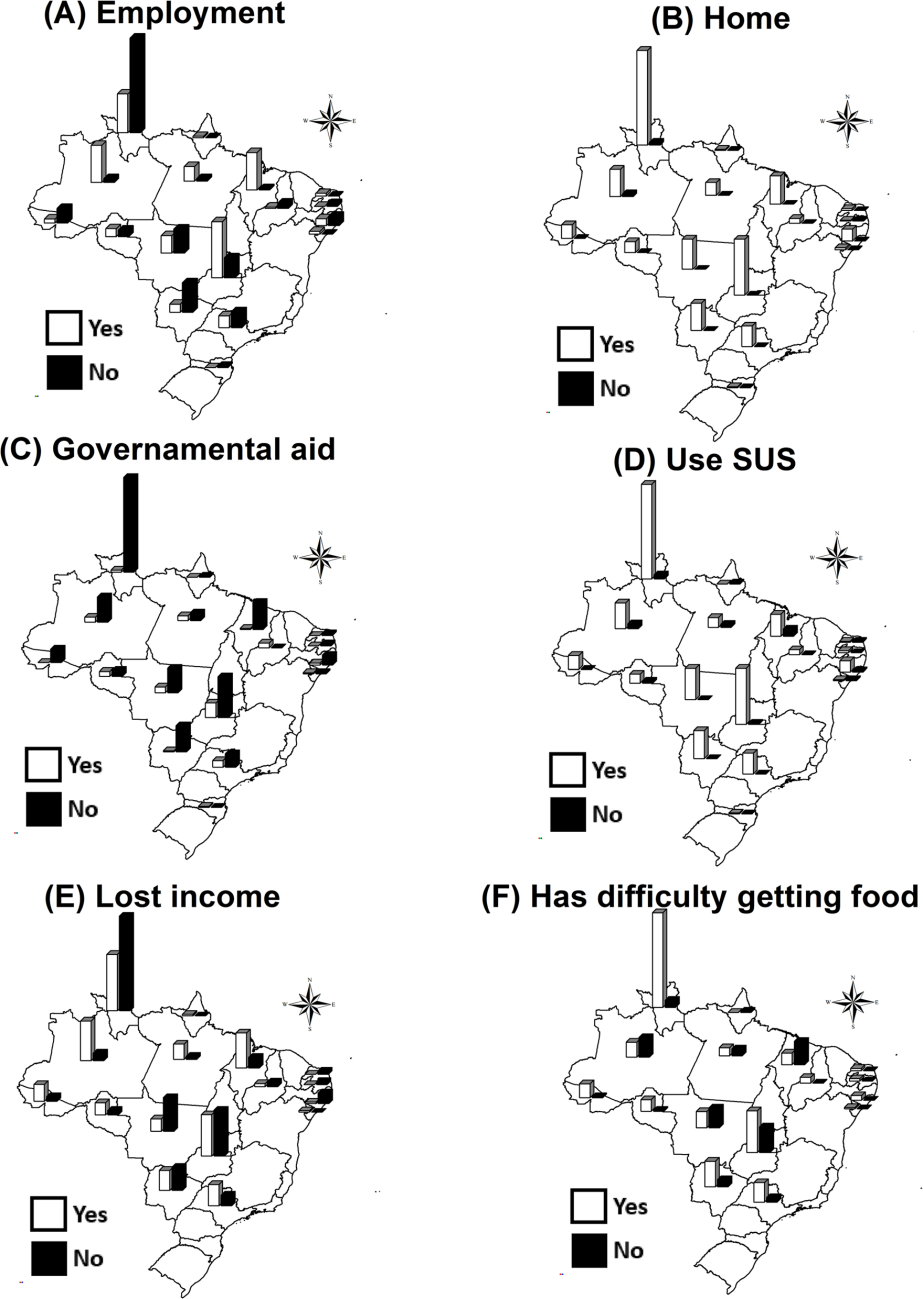
Map of the situation of international migrants and/or refugees regarding employment, housing, government assistance, use of the Unified Health System (SUS), loss of income, and financial difficulty in acquiring food during the COVID-19 pandemic, Brazil (2022–2023)

Of the FU where international migrants and/or refugees were interviewed, it was found that in the Northeast (Alagoas, Paraíba and Piauí), North (Acre and Roraima) and Midwest (Mato Grosso and Mato Grosso do Sul) there was a prevalence of unemployment (Fig. 2A). In all macro-regions there was a prevalence of people who reported having a home (Fig. 2B), not receiving government assistance (Fig. 2C), and using the SUS (Fig. 2D).

Regarding income loss during the pandemic, there was a prevalence in the Northern region (Acre, Amapá, Amazonas, Pará, and Rondônia) (Fig. 2E). As for the financial difficulty to buy food during the pandemic, prevalence was found in the Northeast region (Alagoas, Paraíba, Piauí, Rio Grande do Norte, and Sergipe) and North region (Acre, Pará, Rondônia, and Roraima) (Fig. 2F).

Using binary logistic regression, we found that people of white race/color (OR: 11.59; 95% CI: 3.65–42.94), black/brown (OR: 13.24; 95% CI: 4.76–44.23), yellow (OR: 7.03; 95% CI: 2.12–26.81), those with occupation/employment (OR: 3.52; 95% CI: 2.05–6.13), and those earning less than one minimum wage (OR: 2.70; 95% CI: 1.45–5.17) were more likely to lose income during the COVID-19 pandemic. In contrast, people earning more than three minimum wages (OR: 0.23; 95% CI: 0.05–0.88) were less likely to experience income loss (Table 2).

**Table 2:** Association between sociodemographic variables with loss of income during the COVID-19 pandemic, Brazil (2022–2023)

Variables	*P*-value	OR^a^	95%CI^b^
Aged 18 to 29	0.09	0.62	0.35–1.09
Male	0.13	0.67	0.39- 1.13
White	< 0.01***	**11.59**	**3.65–42.94**
Black/Brown	< 0.01***	**13.24**	**4.76–44.23**
Yellow	< 0.01***	**7.03**	**2.12–26.81**
Have an occupation/employment	< 0.01***	**3.52**	**2.05–6.13**
Secondary incomplete or complete	0.07	1.61	0.96–2.73
Less than 1 minimum wage	< 0.01***	**2.70**	**1.45–5.17**
Above 3 minimum wages	**0.04**	**0.23**	**0.05–0.88**

^a^*OR Odds ratio*

^b^95%CI = 95% Confidence Interval

^***^P-value < 0.01

To validate the model presented in Table 2, it was verified that the accuracy of the model through the area under the ROC curve showed a value of 0.78, Hosmer–Lemeshow test (*p* = 0.24), likelihood ratio (*p* =  < 0.01), CoxSnell (0.23), Nagelkerke (0.30) and McFadden (0.19).

In the second logistic regression, it was identified that people of white race/color (OR: 0.15; 95% CI: 0.02–0.67), black/brown (OR: 0.18; 95% CI: 0.03–0.71), and those with occupation/employment (OR: 0.35; 95% CI: 0.15–0.78) were less likely to have had or to have faced financial difficulty purchasing food during the COVID-19 pandemic. On the other hand, people who declared having no income (OR: 4.03; 95% CI: 1.62–10.26), those who received less than one minimum wage (OR: 2.94; 95% CI: 1.46–6.06), and those who had a confirmed diagnosis of COVID-19 before or after the vaccine (OR: 2.22; 95% CI: 1.04–5.06) were more likely to have experienced financial difficulty purchasing food during the COVID-19 pandemic (Table 3).

**Table 3:** Association of sociodemographic and clinical variables with having had or still having financial difficulty in acquiring food during the pandemic of COVID-19, Brazil (2022–2023)

Variables	*P*-value	OR^a^	95%CI^b^
60 years and older	0.98	NA^c^	NA^c^
Male	0.05	0.55	0.29–1.01
White	**0.02**	**0.15**	**0.02–0.67**
Black/Brown	**0.03**	**0.18**	**0.03–0.71**
Yellow	0.09	0.23	0.03–1.09
Have an occupation/employment	**0.01**	**0.35**	**0.15–0.78**
Has a house	0.99	NA^c^	NA^c^
No schooling	0.17	5.50	0.61–124.54
No income	** < 0.01**	**4.03**	**1.62–10.26**
Less than 1 minimum wage	**0.00**	**2.94**	**1.46–6.06**
Has a chronic disease	0.07	2.60	0.98–7.91
I am more agitated, anxious or tense	0.08	0.55	0.28–1.08
Had a confirmed diagnosis of COVID-19 before or after the vaccination	**0.04**	**2.22**	**1.04–5.06**

^a^*OR Odds ratio*

^b^*95%CI* 95% Confidence Interval

^c^*NA* Not Applicable

For validation of the model presented in Table 3, it was verified that the accuracy capacity of the model through the area under the ROC Curve presented a value of 0.83, Hosmer–Lemeshow test (*p* = 0.59), likelihood ratio (*p* =  < 0.01), CoxSnell (0.28), Nagelkerke (0.39) and McFadden (0.26).

## Discussion

The study sought to survey the social impact of COVID-19 on migrants and refugees regarding income loss, food insecurity, and other social inequities, and to identify explanatory factors on these aspects. Among the origins, we observed that most of the migrants and/or refugees were from sub-Saharan Africa and South America. We also observed a high prevalence of unemployment, income loss and food insecurity, especially in the North and Northeast regions of Brazil.

The literature has highlighted the challenges related to the inclusion of international migrants and/or refugees in public policies [30] and their coverage by the Unified Health System [31]. Evidence shows that Venezuelans are 30% less likely to receive social protection and 64% less likely to be employed [32]. We observed in our results that 25.3% of international migrants and/or refugees lost income partially during the pandemic and 25.3% lost it completely. It was also found that 44.2% already had a pre-existing financial difficulty, which only worsened with the COVID-19 pandemic, demonstrating and confirming how scarce social protection is in this population even before the COVID-19 pandemic.

In the search for the reduction of social inequalities, the Sustainable Development Goals (SDGs) in Brazil include, in goal 10 [33], the task of facilitating orderly, safe, regular and responsible migration and mobility of people, including through the implementation of planned and well-managed migration policies. However, the COVID-19 pandemic has become a challenge for the achievement of the proposed goals, since its closure of international borders to contain the disease, further amplifying the pre-existing inequalities [34].

The flow of international migrants and refugees from sub-Saharan African coast laid bare the vulnerabilities witnessed by this population, from the reduced access to employment and income caused by the need for physical isolation and the countries in South America identified in this study are a reflection of the international scenario, especially where there are crises and conflicts in African countries and the Americas, contributing to the forced migration of populations in situations of social vulnerability and rekindling the concern with food security. In this context, food insecurity emerges as a major consequence of forced international migration, and constitutes an emerging global public health problem, because concomitant with the growing population, displacement also expands the range of diseases [35].

Studies conducted in Canada, the United States of America, Australia, Lebanon, Spain, Thailand, Algeria, and Kenya have highlighted food inequity, cultural adaptation and nutrition, emerging diseases, and nutritional health promotion strategies as implications of forced migration on the food and nutrition of international migrants and refugees [36–49].

Changes in the labor market, the burden of unemployment, violence and inequalities are elements that have historically afflicted Latin American countries [50, 51]. When considering the relationship between migration and health in Brazil, it can be seen that there are challenges related to social policies for the reception and integration of international migrants and refugees considering the globalization process [52]. Thus, it is necessary to promote equity of access to health care, prevention against discrimination, expansion of public policies, training of professionals and provision of adapted services [52].

In this study, the findings identified higher levels of unemployment in the North, Northeast and Midwest regions, with the first two also having greater financial difficulty in acquiring food during the pandemic, and the North region had an even higher rate of income loss. This can be understood by the fact that Brazil is a country of continental dimensions, where the North and Northeast regions suffer from structural, economic and social problems, even before the pandemic, compared to the other regions of the country, and it is known that such problems were exacerbated during COVID-19 [53, 54].

The findings also showed that there was a prevalence in all macro-regions of people who reported not receiving government assistance. However, in Brazil, Article 4, item VIII, of the Migration Law instituted in 2017 [55], reads that social assistance is the right of all international migrants and refugees, regardless of whether their situation is regular, i.e., with a residence permit in the country, or irregular, without a residence permit. Moreover, Law no. 13.982/2020 [56], which established the exceptional measures of social protection due to the COVID-19 pandemic, stated that the concession of the benefit was a fundamental necessary right for groups in vulnerable situations to have their dignity assured, including international migrants and refugees. Despite this context, it is clear that the social protection actions instituted by the Brazilian federative entities faced problems in the execution and implementation of the Emergency Aid, directly harming vulnerable citizens [57].

Taken together, the associations identified between loss of income and financial difficulty to acquire food among the international migrants and refugees reached in this study was complemented by the higher chances of losing income and or having financial difficulty to acquire food during the pandemic in people who had no income and those who received less than one minimum wage, while those who received above three minimum wages the chances were lower. Thus, it is verified how much the migrations and the attention to people in situations of vulnerability related to previous migratory flows and also imposed by the pandemic of COVID-19 must be addressed as social determinants of health and from the perspective of critical epidemiology, because the socioeconomic inequalities of international migrants and refugees expose them to greater situations of vulnerability, illness and lower quality of life [52].

Although the economic crisis hit everyone during the pandemic with unemployment and loss of income, the populations in situations of social vulnerability were certainly the hardest hit. In this context, the results showed that the highest odds of losing income during the pandemic were independent of race/color, and that white and black/brown people were less likely to have had or to have financial difficulty in acquiring food during the pandemic.

However, it is known that black and brown people are more prone to situations of social vulnerability due to structural inequalities in Brazilian society, seen mainly by informal work bonds [58]. In addition, this population is susceptible to other risks, as pointed out in a study [59] in which the average number of deaths by COVID-19 in the five macro-regions of Brazil was higher among black people, which highlights the high degree of exposure of this population to COVID-19, an aspect that is related to several social and structural difficulties, such as the fact that approximately 40% of citizens perform informal work in Brazil [60] and do not have support from labor rights or other sources of income [61], generating a deficit in adherence to physical distancing measures due to the need to remain circulating on the streets in virtue of maintaining the income necessary for survival [62].

The results further highlighted that people who had occupation/employment were more likely to have lost income during the pandemic and were less likely to have had or to have financial difficulty acquiring food during the pandemic. A previous study emphasized that social isolation, along with insecure labor employment ties, especially for international migrants and refugees with regular documentary status, potentiated, during the pandemic, reduced access to food, worsened quality of food intake, reduced number of daily meals, and increased hunger [63].

People who had a confirmed diagnosis of COVID-19 before or after the vaccine had higher odds of having had or having financial difficulty acquiring food during the COVID-19 pandemic. Such a finding may be associated with physical isolation as an individual and collective protection measure. In 2020, the isolation time recommended by the country's health organizations was 14 days after the onset of symptoms [64] for positive cases. This period is equivalent to almost half a month, a very expressive time when considering that people with a positive diagnosis of COVID-19 had to withdraw from work activities, suffering with the impacts on income and, consequently, on food [65].

Although Brazil is known as a happy and hospitable country, international migrants and refugees do not always encounter such a reality. Social inequality, racism, dehumanization, lack of basic rights and their social consequences are recurrent problems in Brazil [66]. Brazil is built by the homogenization of cultures and people of different nationalities, promoted from a historical process of colonization, being marked by conditions inherent to this system that introduced in society social and structural weaknesses, such as prejudice, racism, violence against indigenous peoples, conservatism and public policies failures to ensure the rights of vulnerable populations, among them international migrants and refugees.

Xenophobia can also be a determinant, which occurs in the country in different formats, whether by physical characteristics, culture, cuisine, local language, socioeconomic status, or other aspects that distinguish people [67]. The exercise of epidemiology as a field of isolated knowledge is perceived as unfeasible. It is necessary to think of combined theoretical transdisciplinarity and interculturality and health practice with a transforming character [50, 51], as proposed by critical epidemiology. For this, there is an urgent need for demands for the conformation of methods of participation/community building and health policies that account for the new challenges imposed on vulnerable populations and by contemporary society [50, 51], which are even more perverse in the post-pandemic scenarios of COVID-19.

As the main limitation of this study is the instrument used for the interviews, immigrants were not asked about the length of time they had lived in Brazil and we understand that knowing the length of stay and residence would help to better understand the context of social inequalities, income and food insecurity of this population.

We also mention the non-probabilistic sample by convenience, due to the fact that the interviews took place only in the capital cities of the Brazilian states, limiting the socially vulnerable populations of these territories. However, the approach was used in these areas due to the fact that international migrants and refugees move mainly to the large cities in search of better conditions and opportunities. We also emphasize that some Brazilian capitals were not contemplated and therefore, it is suggested that studies with other approaches aimed at understanding the health of the population of international migrants and refugees be carried out at the national level.

The results presented here, although not generalizable to the reality of all international migrants and/or refugees in Brazil, advance knowledge by contributing with an important portrait of the socio-demographic, clinical and perspectives related to financial conditions of these populations that are still invisible to the eyes of the government and public policies.

In conclusion, the findings highlight the relationship between the sociodemographic characteristics of international migrants and/or refugees with the loss of income and financial difficulty to acquire food during the pandemic. The loss of income presents a strong relation to financial difficulty to acquire food, especially among international migrants and/or refugees who experienced the territorial inequities of Brazil, such as those located in the North and Northeast regions, and those with greater conditions of social vulnerability, who had no income and who received less than a minimum wage.

The inequality underpinned by the historical relationship of oppression and hierarchies in Brazil, imposed by the divisions between social classes, raise the conditions of risk of selectivity of social and environmental protection. In this sense, it is important to highlight that the SDGs presents goals to the population of international migrants and refugees and reaffirms a look at the perspectives related to the orderly and safe conduct in the contribution to economic and social growth, through rights focused on food and social protection.

Strategies for structured actions that consider the peculiarities and socio-political perspectives of international migrants and/or refugees should be encouraged, in order to guide assertive public policies aimed at reducing vulnerabilities. Also, future studies should be based on the movement of understanding the territorial and social reality of international migrants and refugees, in order to consider the context in which they are inserted and, especially, paying attention to the post-pandemic period, where the repercussions may show different realities.

## Data Availability

The datasets used and/or analysed during the current study are available from the corresponding author on reasonable request.

## Abbreviations

COVID-19: Coronavirus Disease 2019
ENSP/UNL: National School of Public Health at the New University of Lisbon
USP: University of São Paulo
EDC: Electronic Data Capture
SM: Social Movements
WHO: World Health Organization
ICF: Informed Consent Form
FU: Federative Units
IBGE: Brazilian Institute of Geography and Statistics *(Instituto Brasileiro de Geografia e Estatística.*)
SUS: Unified Health System (*Sistema Único de Saúde*)
VIF: Variance Inflation Factor
AIC: Akaike Information Criterion
OR: Odds Ratio
95%CI: 95% Confidence Intervals
ROC: Receiver Operating Characteristic
NA: No Answer
NA: Not applicable
SDGs: Sustainable Development Goals
